# NF-κB-related decrease of glioma angiogenic potential by graphite nanoparticles and graphene oxide nanoplatelets

**DOI:** 10.1038/s41598-018-33179-3

**Published:** 2018-10-03

**Authors:** Mateusz Wierzbicki, Ewa Sawosz, Barbara Strojny, Sławomir Jaworski, Marta Grodzik, André Chwalibog

**Affiliations:** 1 Division of Nanobiotechnology, Warsaw University of Life Science, Ciszewskiego 8, 02-786 Warsaw, Poland; 2 Department of Veterinary and Animal Sciences, University of Copenhagen, Groennegaardsvej 3, 1870 Frederiksberg, Denmark

## Abstract

Gliomas develop an expanded vessel network and a microenvironment characterized by an altered redox environment, which produces high levels of reactive oxygen species (ROS) and reactive nitrogen species (RNS) that fuel its growth and malignancy. ROS and RNS can influence tumor cell malignancy via the redox-regulated transcription factor NF-κB, whose activation is further regulated by the mutation status of p53. The objective of this study was to assess the influence of graphite nanoparticles (NG) and graphene oxide nanoplatelets (nGO) on the angiogenic potential of glioma cell lines with different p53 statuses. Nanoparticle treatment of glioma cells decreased the angiogenesis of human umbilical vein endothelial cells (HUVEC) cocultured with U87 (p53 wild type) and was not effective for U118 (p53 mutant) cells. Nanoparticle activity was related to the decreased level of intracellular ROS and RNS, which downregulated NF-κB signaling depending on the p53 status of the cell line. Activation of NF-κB signaling affected downstream protein levels of interleukin 6, interleukin 8, growth-regulated oncogene α, and monocyte chemotactic protein 1. These results indicate that the activity of NG and nGO can be regulated by the mutation status of glioma cells and therefore give new insights into the use of nanoparticles in personalized biomedical applications regarding glioma angiogenesis and its microenvironment.

## Introduction

Gliomas, which are some of the most common malignant tumors of the central nervous system, develop a microenvironment that is characterized by an altered redox state and an abundance of proangiogenic and proinflammatory factors^1^. Gliomas develop an expanded vessels network and angiogenesis pathologies including vascular hyperproliferation and hemorrhage caused by the breakdown of the intratumoral blood–brain barrier^2^. Proangiogenic signals in tumors are fueled by cycling hypoxia, ROS, RNS, acidosis, and inflammation^1,3^. Tumor cells, including gliomas, maintain an altered redox environment with high production of ROS and RNS that causes tumorigenic cell signaling^4^. One main source of ROS in tumor cells is the NADPH oxidase family, which are plasma membrane-bound enzymes that produce superoxide through single-electron reduction^5^. Nitric oxide is produced by nitric oxide synthase (NOS), which forms the second most common RNS, peroxynitrite, after reacting with superoxide^6^. ROS and RNS influence tumor cell malignancy in different ways, but one of the most important is regulation of NF-κB transcription factor activation. NF-κB regulates numerous genes, including those involved in the development of the tumor microenvironment and the synthesis of proangiogenic and proinflammatory cytokines^7^. NF-κB activation is also regulated by the mutation status of the tumor suppressor, p53^8^. p53 is one of the most frequently mutated genes due to its potent antitumor activities. Mutations in p53 lead to the inhibition of its principal activity, tumor suppression. Moreover, tumors with p53 mutations often show gain-of-function phenotypes that usually enhance their malignancy, including enhanced invasiveness and decreased sensitivity to proapoptotic signals^9^. Gain-of-function phenotypes originate from the increased half-life of p53, which influences signaling pathways in tumor cells and increases genomic instability^10^.

Carbon nanoparticles exert a redox-modulating property that originates from their unique structure and the localization of functional groups on their surface. The occurrence of numerous oxygen-containing functional groups on carbon nanoparticles, such as the close proximity of carboxyl and hydroxyl groups, enables them to act as reducing agents^11^. Graphene oxide and other graphene-based materials are effective scavengers of hydroxyl radicals and superoxide and can have properties of a weak H-donor antioxidant^12^. Graphite nanoparticles (NG) have a similar structure to graphene, thus their antioxidant properties should not differ greatly. Due to the intensive endocytosis of NG and graphene oxide nanoplatelets (nGO) by glioma cells, it is hypothesized that nGO and NG will decrease intracellular ROS^13^. Moreover, it is assumed that this will decrease NF-κB-dependent proangiogenic cytokines in a p53 wild-type glioma cell line (U87) but not in a p53 mutant cell line (U118).

## Results

### NG and nGO change the angiogenic potential of U87 but not U118 glioma cell lines

**Figure 1:**
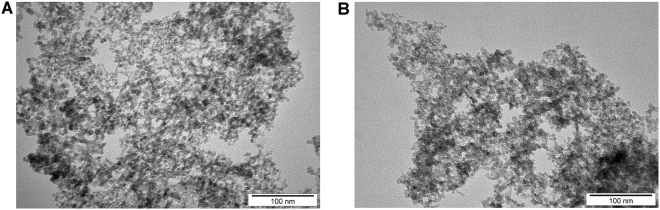
Nanoparticle morphology. Transmission electron microscopy images of (**A**) graphite nanoparticles and (**B**) graphene oxide nanoplatelets.

The physicochemical properties of NG and nGO were initially confirmed by investigating the nanoparticles using transmission electron microscopy (TEM) and analyzing their zeta potential. The Raman spectra of analyzed nanoparticles were recently published^13^. TEM images were used to confirm the nanoparticle morphology (Fig. 1); NG were spherical nanoparticles of approximately 8 nm, whereas nGO were of similar size and had a platelet morphology due to the method of synthesis from NG. The zeta potential was analyzed to characterize surface charges and the stability of the suspensions. The zeta potential of NG and nGO were 40.1 and 20.3 mV respectively, showing more stable hydrocolloids in NG.

**Figure 2:**
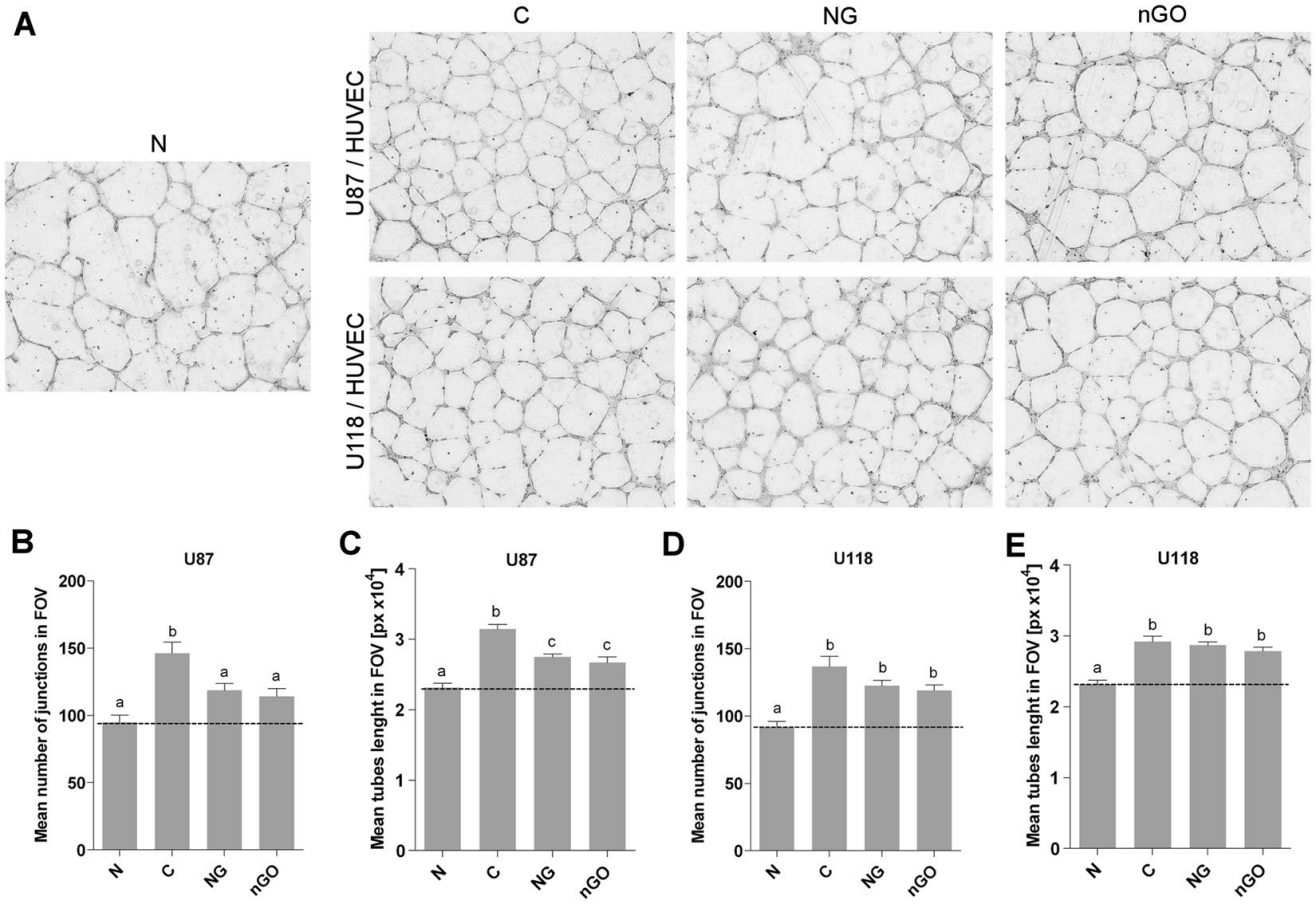
NG and nGO decrease the angiogenic properties of U87 but not U118 glioma cells. Angiogenic properties were examined using indirect coculture (inserts) of glioma cell lines U87 and U118 with HUVEC on a thin layer of extracellular matrix. (**A**) Images of HUVEC tube formation following treatment with or without (C; control) graphite nanoparticles (NG) and graphene oxide nanoplatelets (nGO) in U87 or U118 cells in an insert above the HUVEC. The negative control (N) was conducted by culturing HUVEC with an insert but without glioma cells. Graphs show the mean number of junctions of HUVEC tubes in the field of view after coculture with U87 (**B**) and U118 (**D**) cells and the mean tube length in the field of view after coculture with U87 (**C**) and U118 (**E**) cells. Data were obtained by analyzing images using ImageJ software and the Angiogenesis Analyzer macro. Values are expressed as mean ± standard deviation. Statistical significance is indicated with different superscripts (one-way ANOVA; *P* < 0.05). *FOV*, field of view; *px*, pixels.

Analysis of human umbilical vein endothelial cells (HUVEC) tube formation in coculture with U87 (p53 wild type) glioma cell line treated with NG or nGO decreased the examined angiogenesis parameters (Fig. 2). The total tube length and the number of junctions in HUVEC were used to indicate the angiogenic potential of glioma cells. Neither NG nor nGO treatment of U118 cells (with p53 mutations) changed the assessed HUVEC angiogenesis parameters.

### NG and nGO have limited toxicity in glioma cells

**Figure 3:**
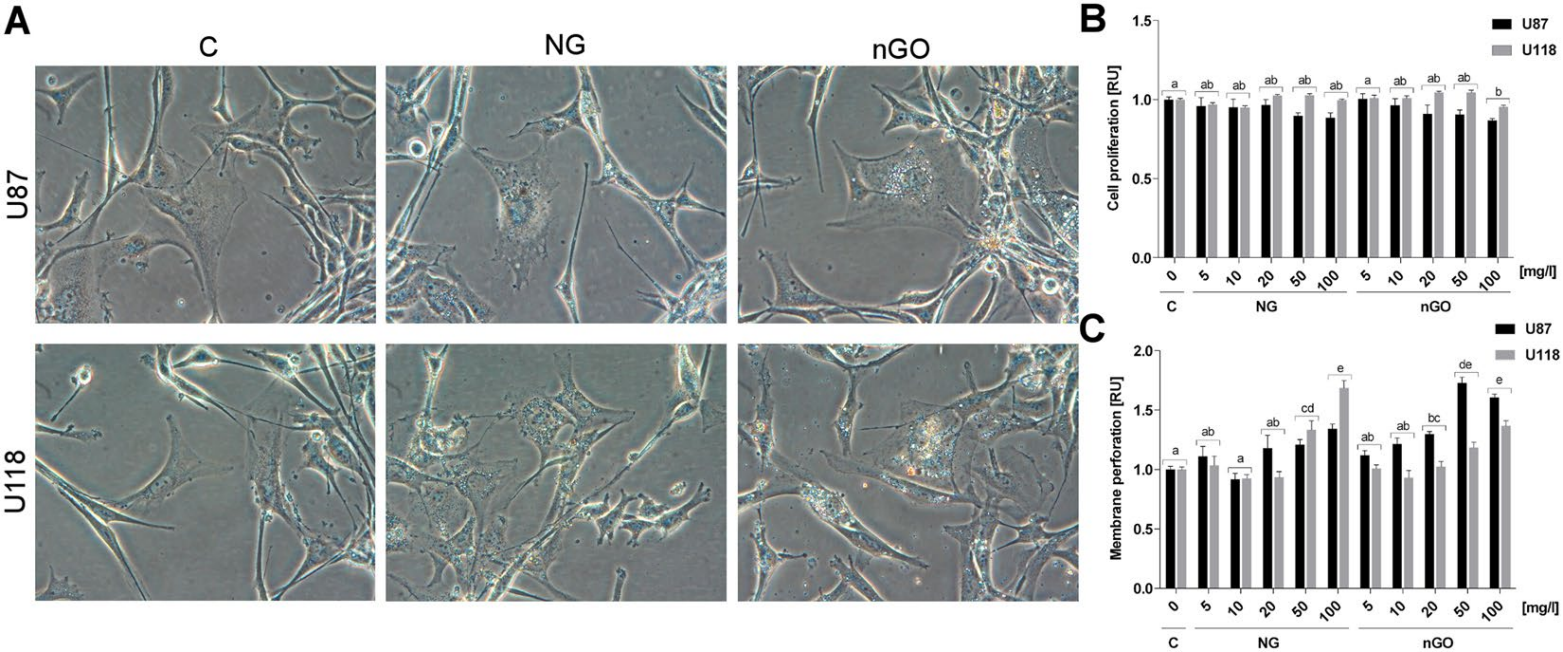
NG and nGO have limited toxicity in glioma cells. (**A**) Morphology of U87 and U118 glioma cells with or without treatment (C; control) with graphite nanoparticles (NG) and graphene oxide nanoplatelets (nGO). Morphology was assessed by light microscopy using phase contrast with 400X magnification. (**B**) Cell proliferation and (**C**) membrane perforation were determined using LDH assays. Cells were exposed to NG and nGO at concentrations of 5, 10, 20, 50, and 100 μg/ml for 24 h. Statistical significance is indicated with different superscripts (multifactor ANOVA; *P* < 0.05). *RU*, relative units.

Proliferation and membrane perforation were investigated using an lactate dehydrogenase (LDH) assay to evaluate if the observed decrease in the angiogenic potential of U87 glioma cells following NG or nGO treatment was not related with the direct toxicity of those nanomaterials (Fig. 3). NG and nGO were added to cell cultures of U87 and U118 cell lines at concentrations of 5, 10, 20, 50, and 100 μg/ml. NG did not influence the proliferation of glioma cells; however, nGO decreased the proliferation but only after treatment with 100 μg/ml. There were also significant differences between the cell lines (*P* = 0.0000) and in the interactions between the cell lines and the nanoparticles (*P* = 0.0124). The highest concentrations of NG and nGO also increased membrane perforation. Similar to the proliferation assay, there was a significant difference between the cell lines (*P* = 0.0000) and in the interactions between the cell lines and nanoparticles (*P* = 0.0000). Cell morphology was also investigated (Fig. 3A), and nanoparticle treatment resulted in the formation of light-reflecting structures inside the cells that were probably nanoparticle agglomerates, as previously shown using TEM analysis^13^.

### Treatment with NG and nGO decreases the activation of NF-κB signaling in U87 cells

**Figure 4:**
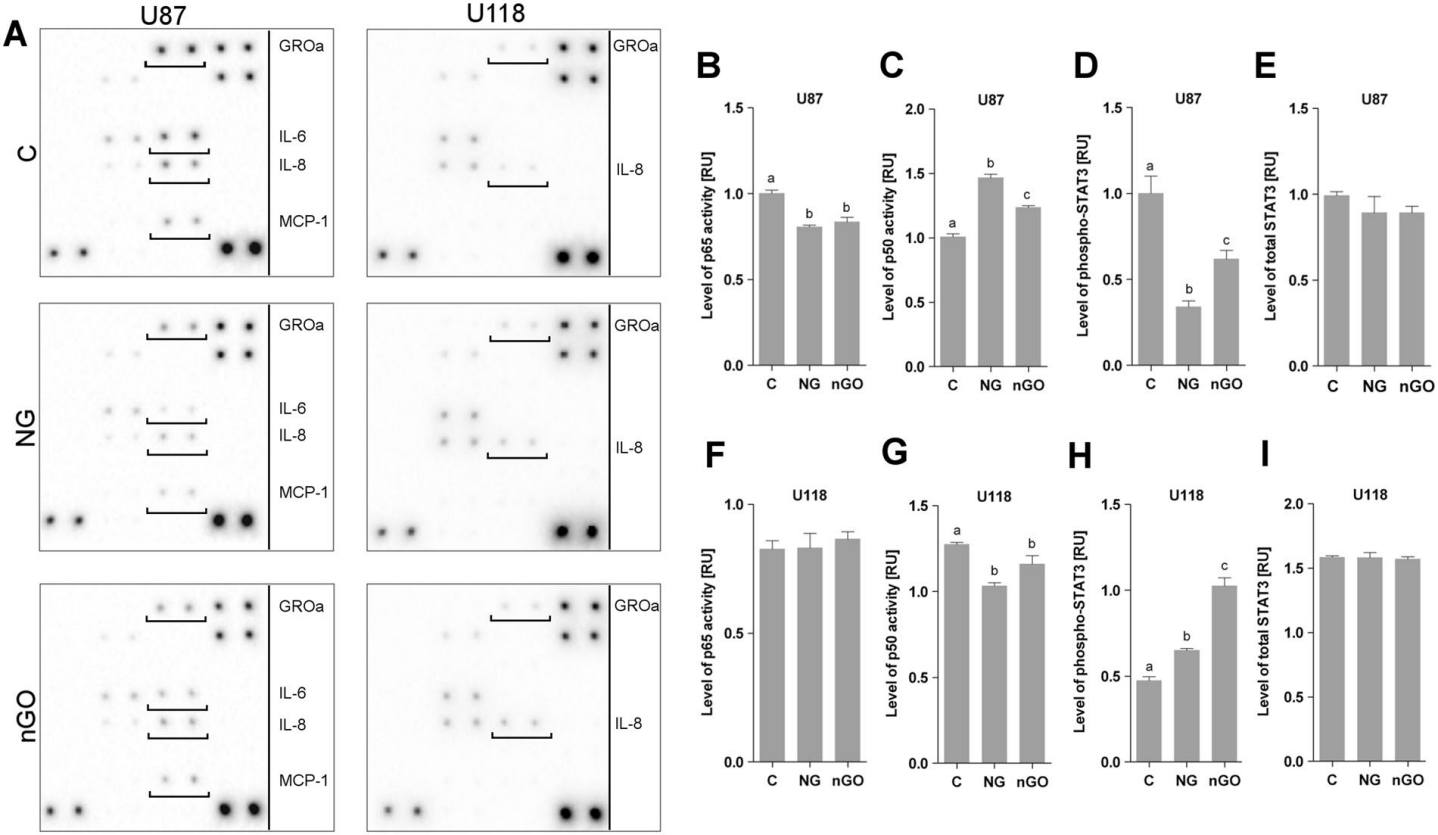
NG and nGO decrease the synthesis of NF-κB related proteins. (**A**) Antibody array analysis of proangiogenic cytokine synthesis in U87 and U118 glioma cells with or without treatment (C; control) with graphite nanoparticles (NG) and graphene oxide nanoplatelets (nGO). The full array map and uncropped images are available in the supplement. Nanoparticle treatment results in decreased synthesis of GROα, IL-6, IL-8, and MCP-1 in U87 but not U118 cells. Transcription factor assay analysis of the activity of p50 and p65 NF-κB subunits in the nuclear fraction of U87 (**B**,**C**) and U118 (**F**,**G**) cells. Nanoparticle treatment of U87 cells decreased the activation of the p65 subunit and increased the activation of the p50 subunit. Analysis of STAT3 activation showed decreased STAT3 phosphorylation after nanoparticle treatment of U87 cells (**D**) but increased phosphorylation after treatment of U118 cells (**H**). The total STAT3 protein level was not changed (**E**,**I**). Statistical significance is indicated with different superscripts (one-way ANOVA; *P* < 0.05). *RU*, relative units.

To understand the phenomenon of decreased angiogenic activity in the U87 glioma cell line after NG and nGO treatment, 20 cytokines that are important in angiogenesis were analyzed, including vascular endothelial growth factor A (VEGF-A), basic fibroblast growth factor (bFGF), interleukin 6 and 8 (IL-6 and IL-8), growth-regulated oncogene α (GROα; CXCL1), and monocyte chemotactic protein 1 (MCP-1) (an array map with a list of all analyzed cytokines and uncropped images are included in Supplemental Figs S1–S3). In U87 cells, nanoparticles did not affect the levels of the most potent proangiogenic factors (i.e., VEGF-A and bFGF) but decreased the synthesis of IL-6, IL-8, GROα (CXCL1), and MCP-1 (Fig. 4A). The U118 cell line showed only a minor increase in the synthesis of IL-8.

IL-6 activates signal transducer and activator of transcription 3 (STAT3), thus to confirm the changes in IL-6 protein levels after NG and nGO treatment, the phosphorylation level of STAT3 was assessed. Treatment of U87 cells decreased the STAT3 phosphorylation level without changing the total STAT3 protein level (Fig. 4D and E). Conversely, nanoparticles increased the STAT3 phosphorylation level in U118-treated cells (Fig. 4H and I).

NF-κB is a common regulator of IL-6, IL-8, GROα, and MCP-1 thus the binding activity of NF-κB subunits p50 and p65 to DNA-containing NF-κB response elements was assessed. The binding activity of the p65 subunit after nanoparticle treatment of U87 cells was significantly decreased, thus both NG and nGO reduced NF-κB signaling. In addition, the activity of the p50 subunit was also increased. Nanoparticles did not affect the p65 subunit in U118 cells but decreased the binding activity of the p50 subunit.

### NG and nGO decrease the levels of intracellular ROS and nitric oxide

**Figure 5:**
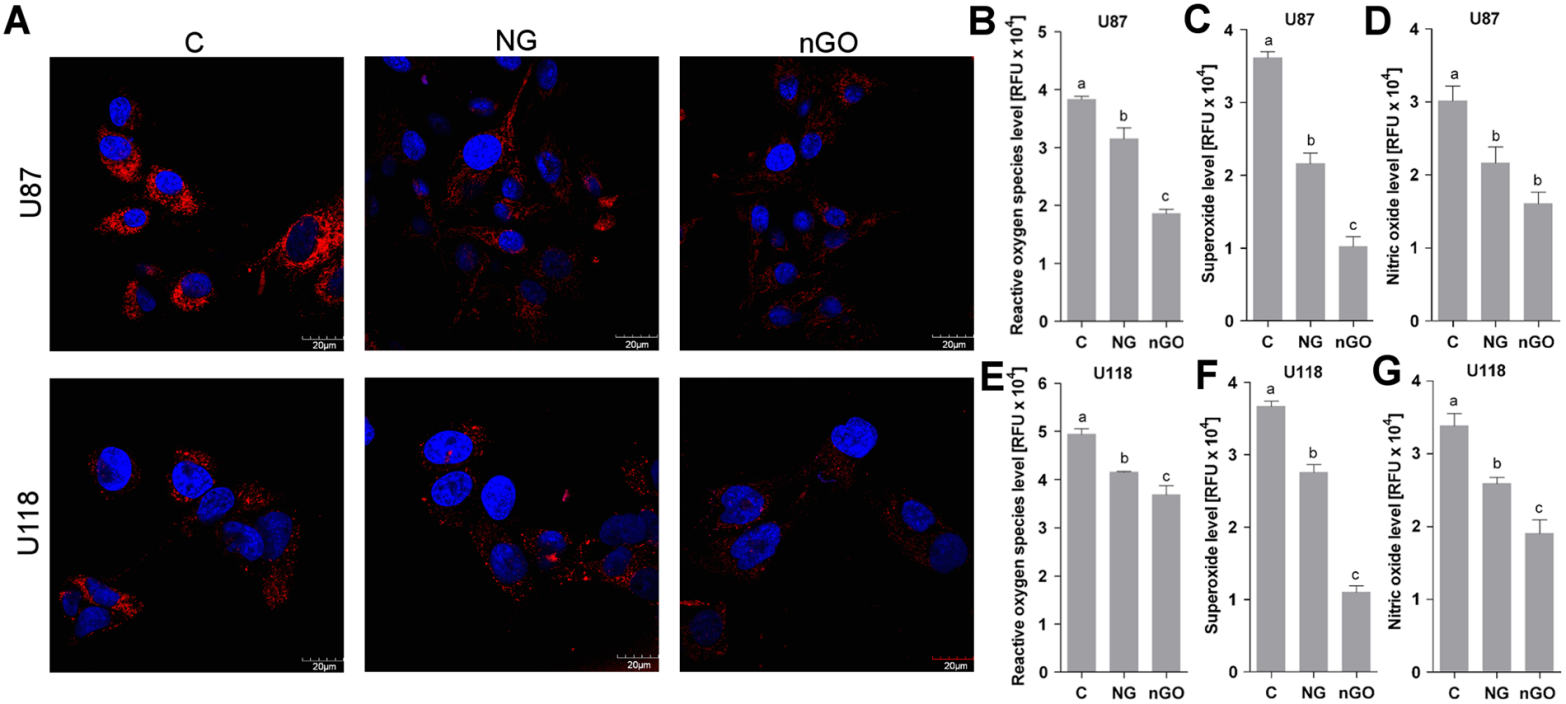
NG and nGO treatment decrease ROS and nitric oxide levels. (**A**) Confocal microscopy analysis of superoxide synthesis in U87 and U118 glioma cells with or without treatment (C; control) with graphite nanoparticles (NG) and graphene oxide nanoplatelets (nGO). MitoSOX reagent oxidized by superoxide exerts fluorescence in mitochondria (red color); nuclei are counterstained with Hoechst 33342. Nanoparticle treatment decreased superoxide levels in both cell lines. Similar results were obtained in the microplate analysis of superoxide levels (**C**,**F**). Analysis of total ROS levels using CellROX reagent in U87 (**B**) and U118 (**E**) cells showed a decrease in ROS after nanoparticle treatment. Analysis of nitric oxide levels in U87 (**D**) and U118 (**G**) cells similarly showed a decrease in nitric oxide after nanoparticle treatment. Statistical significance is indicated with different superscripts (one-way ANOVA; *P* < 0.05). *RFU*, relative fluorescence units.

Intracellular synthesis of ROS and nitric oxide were analyzed to understand the reason behind the decreased NF-κB signaling in U87 glioma cells treated with NG and nGO. ROS synthesis was assessed using two tests: the first for total ROS analysis and the second for determining the mitochondrial superoxide level. Treatment of U87 glioma cells with NG and nGO decreased the total ROS and, to a greater extent, the mitochondrial superoxide level (Fig. 5B and C). These results were confirmed by confocal microscopy analysis of superoxide levels in glioma cells (Fig. 5A). Furthermore, nitric oxide synthesis was also decreased (Fig. 5D). Similar results were obtained for the U118 cell line, thus showing that the differences in the effectiveness of NG and nGO on angiogenic potential were not related to ROS or nitric oxide levels but to the activity of the NF-κB signaling pathway.

## Discussion

Previous studies showed that carbon nanoparticles were not only intensively taken up by glioma cells but that they also decreased migration and invasiveness due to impaired extracellular adhesion and EGFR/AKT/mTOR signaling pathway regulation.^13^ Therefore it was suggested that carbon nanoparticles (i.e., NG and nGO) could influence other basic physiological activities of glioma cells and that the process of blood vessel growth toward the tumor should be assessed. This study has shown, for the first time, that NG and nGO can influence the angiogenic potential of glioma cells and that the response of glioma cells to carbon nanoparticle treatment can be dependent on p53 status related to NF-κB activity.

Angiogenesis is one of the most important processes during tumor progression. The synthesis of proangiogenic and proinflammatory cytokines leads to the formation of highly vascularized tumors with a characteristic microenvironment. The findings reported here show that two allotropic forms of carbon nanoparticles (NG and nGO) can influence tumor angiogenesis despite their low toxicity, which is characteristic of most carbon nanoparticles^14–16^. Changes in proliferation and membrane perforation were limited, especially for nanoparticles at a final concentration of 20 μg/ml, therefore it was assumed that nanoparticle toxicity was not responsible for the decreased angiogenic potential of U87 glioma cells. The decrease in angiogenesis caused by carbon nanoparticles was previously reported but never in relation to tumor intracellular ROS. Experiments regarding the antiangiogenic properties of carbon nanoparticles showed that among diamond nanoparticles, NG, multiwall nanotubes, fullerene C60, and pristine graphene, the first two had the strongest antiangiogenic activity^17,18^. In addition, multiwall nanotubes inhibited angiogenesis, as analyzed using the HUVEC angiogenesis model^19^ and chorioallantoic membrane model, induced by VEGF-A or bFGF^20^.

ROS promote tumor cell angiogenesis by several pathways including NF-κB activation. In addition, ROS can promote angiogenesis by stabilizing hypoxia-inducible factors (HIF) and activating 5′-adenosine monophosphate-activated protein kinase^21^. NF-κB is a transcription factor that regulates the expression of multiple genes and different cellular functions (i.e., inflammation and immunity); however, it is also known to regulate angiogenesis due to the increased synthesis of several pro-angiogenic proteins, including IL-6 and IL-8^22^. NF-κB proteins consist of different family members, including p65, that homo- or heterodimerize and have a C-terminal transcription activation domain that is essential for DNA binding and positive gene regulation. On the other hand, p50 can bind to DNA NF-κB binding sites but it cannot activate transcription unless it forms a heterodimer with p65. Moreover, due to it taking up the binding site on DNA, the p50 homodimer is considered as a competitive inhibitor of the NF-κB signaling pathway^23^. Excess p50 downregulates p65, thus suggesting that a p50 homodimer may modulate transcription in place of the p50–p65 heterodimer that activates transcription of NF-κB-related genes^24^.

ROS regulation of the NF-κB signaling pathway is complicated and depends on several processes; however, the main direct mechanisms of regulation consist of phosphorylation and direct oxidation of NF-κB subunits^7,25^. In this study, decreased intracellular ROS increased the p50 subunit activation and decreased p65 subunit activation, which is in accordance with results of p50 and p65 regulation by ROS. The transcriptional activity of p65 is dependent on serine phosphorylation (Ser-276), which determinates its interaction with transcriptional coactivators. Phosphorylation of Ser-276 depends on ROS, and antioxidants have been shown to decrease Ser-276 phosphorylation^26–28^. The regulation of p50 function by ROS is different to that reported for the p65 subunit. Oxidation of p50 by ROS inhibits its DNA binding ability due to the oxidative sensitivity of a key cysteine (Cys-62) in the transcription activation domain^29,30^. In addition, this cysteine may also be S-nitrosylated by nitric oxide^31^, which was decreased. Interestingly, inducible NOS is upregulated by the NF-κB signaling pathway^32^. Thus the observed decrease in nitric oxide synthesis could originate from both the activity of NG and nGO and the downregulation of the NF-κB signaling pathway. However, the scavenging activity of nanoparticles is more probable due to the observed decrease in nitric oxide synthesis in both cell lines. Consequently, p65 activity after nanoparticle treatment was not changed. Moreover, the results showing nGO scavenging activity were in accordance with other research showing that graphene oxide and other graphene-based materials were effective scavengers of hydroxyl radicals and superoxide and had properties of weak H-donor antioxidants^12^.

These results showed that NF-κB activation and its downstream effects are dependent on the p53 status of glioma cell lines. Wild-type p53 in the U87 cell line seemed to be responsible for the effectiveness of NG and nGO in decreasing NF-κB signaling activation. U87 and U118 cell lines are often used as p53 wild-type and mutant glioma cell lines, respectively, due to the presence of phosphatase and tensin homolog mutations in both cell lines^33–35^. p53 mutations activate NF-κB signaling, and transfection of wild-type p53 into p53-null lung cancer cell lines suppressed nuclear translocation of p65^36^. Moreover, promotion and prolongation of NF-κB signaling by a p53 mutant was described as one gain-of-function mechanism that leads to chronic inflammation^8,9^.

NF-κB activation after NG and nGO treatment decreased the synthesis of IL-6, IL-8, GROα, and MCP-1. The decreased synthesis of those cytokines in U87 glioma cells diminished the angiogenic properties, which were analyzed using indirect coculture of glioma cells and HUVEC. GROα is a chemoattractant molecule and a growth factor that induces angiogenesis and increases the tumorigenic potential of glioma cells^37^. Conversely, in addition to MCP-1 being a strong monocyte chemoattractant, it is also a potent angiogenic factor that stimulates HIF-1α and VEGF synthesis through a specific transcription factor (MCP-1-induced protein)^38^. The regulation of IL-6, IL-8, GROα, and MCP-1 synthesis reported here is often reported in studies concerning the activation of the NF-κB signaling pathway^22,39^. This suggests that the regulation of NF-κB signaling pathways by NG and nGO treatment was responsible for the observed results. Moreover, IL-6 is considered to be one of the most highly induced NF-κB-dependent cytokines^40^. IL-6 is an inflammatory cytokine that not only regulates the immune inflammatory response but also plays an important role in cell proliferation and angiogenesis. Due to it pleiotropic activity, IL-6 is often synthesized by tumor cells, which leads to increased tumorigenesis and the formation of a tumor microenvironment that further enhances malignancy and decreases the effectiveness of therapies^41^. Similarly, IL-8 is a proangiogenic cytokine that exerts proangiogenic activities in many tumors, including glioblastomas^42^. Glioblastomas usually produce several proangiogenic cytokines including VEGF, IL-8, and IL-6. The combined inhibition of both VEGF and IL-6 was proposed to have a promising antitumor effect, and knockdown of both IL-6 and VEGF in a mouse model inhibited tumor development and cell infiltration^43^. Both IL-6 and IL-8 affect several critical angiogenesis processes such as the promotion of endothelial cell migration and proliferation^44,45^. Moreover, IL-6 induces VEGF synthesis in endothelial cells, probably via the activation of hypoxia-induced genes. The VEGF expression level after IL-6 induction was similar to the effect of hypoxia or chemically-activated HIF-1α^46^. The change in IL-6 synthesis following nanoparticle treatment was confirmed by analyzing STAT3 phosphorylation. IL-6 signal transduction leads to Janus kinase (JAK) activation, which mediates STAT3 phosphorylation^47^. The results presented here showed that NG and nGO decreased the IL-6 protein level and STAT3 activation. Several studies analyzing the effects of intracellular ROS, including mitochondrial superoxide, on IL-6 and the NF-κB pathway have also observed major changes in STAT3 activation^48,49^.

These results demonstrate that the antiangiogenic activities of NG and nGO depend on the p53 status and NF-κB regulation. Furthermore, NG and nGO effectively decrease the angiogenic potential of a wild-type p53 glioma cell line (U87) by decreasing intracellular ROS and nitric oxide synthesis and leading to the downregulation of NF-κB-dependent proteins IL-6, IL-8, GROα, and MCP-1. These findings give new insights into the use of NG and nGO in personalized biomedical applications regarding glioma angiogenesis and its microenvironment.

## Material and Methods

### Nanomaterials

NG were purchased from SkySpring Nanomaterials (Houston, USA), while nGO were prepared at the Institute of Electronic Materials Technology from NG through a modified Hummers’ method as previously described^14^. The nanopowders were dispersed in ultrapure water to prepare 1 mg/ml solutions. Immediately prior to cell exposure, hydrocolloids of nanoparticles were sonicated for 30 min and diluted to different concentrations with supplemented Dulbecco’s modified Eagle’s medium (DMEM) (Thermo Fisher Scientific, Waltham, USA).

TEM images of nanoparticles were acquired using a JEM-1220 microscope (Jeol, Tokyo, Japan) at 80 kV with a Morada 11-megapixel camera (Olympus Soft Imaging Solutions, Münster, Germany). Samples were prepared by placing droplets of hydrocolloids onto formvar-coated copper grids (Agar Scientific, Stansted, UK) and air drying before observations.

Zeta potential measurements were carried out with the Nano-ZS90 Zetasizer (Malvern, Worcestershire, United Kingdom) at 25 °C using the Smoluchowski approximation. Each sample was measured after 120 s of stabilization at 25 °C (20 replicates). Nanoparticles were also examined by Raman spectroscopy using an inVia Raman Microscope (Renishaw, Gloucestershire, United Kingdom) with an Nd:YAG 532 nm laser. Hydrocolloids of nanoparticles were placed on a silicon substrate and incubated at 50 °C for 24 h to evaporate water.

### Cell lines

Human glioma cell lines, U87 (p53 wild type) and U118 (p53 mutant), were obtained from the American Type Culture Collection (Manassas, USA) and maintained in DMEM (Thermo Fisher Scientific) supplemented with 10% fetal bovine serum (Thermo Fisher Scientific) and 1% penicillin/streptomycin (Thermo Fisher Scientific). HUVEC were obtained from Thermo Fisher Scientific and maintained in Medium 200 basal media supplemented with a large vessel endothelial supplement (Thermo Fisher Scientific) and 1% penicillin/streptomycin (Thermo Fisher Scientific). Cells were maintained at 37 °C in a humidified atmosphere of 5% CO_2_ and 95% air.

### Angiogenesis potential of glioma cells

The angiogenesis potential of glioma cells was analyzed with a tube formation assay using indirect coculture of U87 or U118 glioma cells with HUVEC. Glioma cells were cultured on a six-well plate 0.4 μm high pore density insert (Corning, New York, USA), and HUVEC were cultured below the insert on a layer of Geltrex Reduced Growth Factor Basement Membrane Matrix (Thermo Scientific). Glioma cells were seeded at a density of 3 × 10^4^ cells/well and incubated for 24 h in supplemented DMEM media. New media composed from supplemented DMEM and nonsupplemented Medium 200 (1:1) with or without (control group) 20 μg/ml of nanoparticles was subsequently introduced to the cells for the next 24 h. In addition, the negative control group (insert without cells) was treated as a control group. After incubation, inserts with glioma cells were placed above HUVEC (seeded at a density of 9.5 × 10^4^ cells/well) in supplemented Medium 200 diluted 2.5-fold with nonsupplemented Medium 200. After 12 h of incubation, images of HUVEC tubes were made using a reversed microscope equipped with a 4X objective using phase contrast. The number of junctions and total tube length were analyzed with ImageJ software^50^ and the Angiogenesis Analyzer macro^51^.

### Cell proliferation and membrane permeabilization using the LDH assay

Cell viability was evaluated using an LDH-based assay kit (Thermo Fisher Scientific). U87 and U118 cells were plated in 96-well plates (5 × 10^3^ cells/well) and incubated for 24 h. New medium containing NG or nGO was introduced to the cells at concentrations of 5, 10, 20, 50, and 100 μg/ml. For cell proliferation analysis, cells were incubated with nanoparticles for 48 h and subsequently lysed for 45 min at 37 °C with lysis buffer. The total amount of LDH (depending on the number of cells) was analyzed by incubation with the reaction mixture at room temperature for 30 min. After the addition of a stop solution, absorbance was analyzed at 490 nm and 680 nm was used as a reference using a Tecan Infinite 200 microplate reader (Tecan, Durham, USA). Cell proliferation was expressed as a relative value after subtracting the absorbance from blank samples. For cell membrane perforation analysis, cells were incubated with nanoparticles for 24 h. The 96-well plates were centrifuged (200 × g; 6 min), and 50 μl of cell medium from each well was transferred to a new 96-well plate. The reaction mixture was subsequently added to each well and incubated for 30 min. The amount of LDH released from the cells was analyzed by the addition of a stop solution, and absorbance was read at 490 nm and 680 nm was used as a reference (Tecan Infinite 200 microplate reader, Tecan). Cell membrane perforation was expressed as a relative value after subtracting the absorbance from blank samples.

### Sample preparation for protein analysis

For protein analysis, glioma cells were treated with NG or nGO at a concentration of 20 μg/ml and incubated for 24 h following a phosphate buffered saline (PBS) wash. Cells not treated with nanoparticles were used as the control. Whole-cell protein extracts were prepared by suspending cells in ice-cold radioimmunoprecipitation assay buffer (RIPA) buffer containing protease and phosphatase inhibitors (Sigma-Aldrich, St. Louis, USA). The cells were incubated on ice for 40 min (vortexing at 10 min intervals) before being centrifuged (30 min; 14,000 × g; 4 °C) and the supernatant collected. The nuclear fraction was obtained by suspending cells in hypotonic buffer (20 mM Tris-HCl, pH 7.4; 10 mM NaCl; 3 mM MgCl_2_). Igepal CA-630 (Sigma-Aldrich) containing protease and phosphatase inhibitors (Sigma-Aldrich) was added to a final concentration of 0.5%, and the solution was vortexed for 10 s. The pellet containing the nuclear fraction was resuspended in ice-cold RIPA buffer containing protease and phosphatase inhibitors and incubated on ice for 30 min (vortexing at 10 min intervals). The supernatant of the nuclear fraction homogenate was collected after centrifugation (30 min; 14,000 × g; 4 °C). Protein concentration was determined using a Bicinchoninic Acid Kit (Sigma-Aldrich).

### Analysis of NF-κB subunit (p65 and p50) activity

NF-κB analyses were carried out using the NF-κB p65 and p50 Transcription Factor Assay Kit (Abcam, Cambridge, United Kingdom) that consists of double-stranded DNA sequence containing the NF-κB response element immobilized onto the bottom of wells. p65 or p50 that was bound to the NF-κB response element was detected by colorimetric readout using anti-p65 or anti-p50 primary antibodies and a horseradish peroxidase (HRP) conjugated secondary antibody. Analyses were performed in accordance with the manufacturer’s instructions using lysates containing 15 μg of nuclear extract protein per well. Experiments were repeated three times.

### Antibody array analysis

Analysis of proangiogenic cytokine synthesis was performed using an antibody array (ab134000; Abcam). The assay was performed in accordance with the manufacturer’s instructions using lysates containing 100 μg/ml of total protein per membrane and cell extracts from three separate experiments. Membranes were visualized using the ChemiDoc Imaging System (Bio-Rad, Hercules, USA).

### STAT3 activation

Levels of total STAT3 protein and STAT3 phosphorylation (p-Y705) were assayed using Enzyme-linked immunosorbent assay (ELISAs) (ab176655 and ab176654; Abcam). ELISAs were performed in accordance with the manufacturer’s instructions using lysates containing 100 μg/ml of total protein. A standard curve was constructed for each assay using serial dilutions of the control lysates. All experiments were repeated twice using cell extracts from three separate experiments.

### ROS and nitric oxide synthesis analysis

Total ROS synthesis in glioma cells was analyzed using CellROX Green Reagent (Thermo Fisher Scientific), while mitochondrial superoxide levels were assessed using MitoSOX Red (Thermo Fisher Scientific). U87 and U118 cells were plated in black 96-well plates (1 × 10^4^ cells/well) and 35 mm glass-bottomed dishes (1 × 10^5^ cells/well; Nest Biotechnology, Wuxi, China) for confocal microscopy analysis and incubated for 24 h. NG or nGO was introduced to the cells at concentrations of 20 μg/ml and incubated for 2 h at 37 °C. Cells were subsequently incubated with CellROX Green Reagent (30 min; 37 °C) at a final concentration of 5 μM in supplemented DMEM medium or MitoSOX Red (10 min; 37 °C) at a final concentration of 5 μM in PBS. Fluorescence was analyzed using a microplate reader (Tecan) after washing with PBS. Quantification of the data was performed by subtracting the fluorescence intensity of blank wells containing cell medium (for the control group) or medium with NG or nGO nanoparticles (for treatment groups). MitoSOX Red staining was also observed using a confocal microscope (FV-1000; Olympus Corporation, Tokyo, Japan) equipped with a temperature- and atmosphere-controlled chamber (37 °C; 5% CO_2_). After the staining procedure described above, cells were counterstained with Hoechst 33342 (Thermo Fisher Scientific) at a final concentration of 10 μM in PBS for 10 min. For visualization using confocal microscopy, cells were kept in PBS supplemented with 1% fetal bovine serum (FBS) (Thermo Fisher Scientific).

Nitric oxide synthesis was determined using a fluorometric kit (Nitric Oxide Synthase Detection System; Sigma-Aldrich). U87 and U118 cells were plated in black 96-well plates (5 × 10^3^ cells/well) and incubated for 24 h. New medium containing NG or nGO was subsequently introduced to the cells at concentrations of 20 μg/ml and incubated for 24 h at 37 °C. Cells were incubated with a reaction mixture containing a diacetate derivative of 4,5-diaminofluorescein at a final concentration of 2.5 μM for 2 h at room temperature and washed with PBS. The fluorescence of triazolo-fluorescein was analyzed using a microplate reader (Tecan). Quantification of the data was performed by subtracting the fluorescence intensity of blank wells containing cell medium (for the control group) or medium with NG or nGO nanoparticles (for treatment groups).

### Statistical analysis

Data were analyzed using one-way and multifactorial analysis of variance with Statgraphics Centurion XVI (StatPoint Technologies, Warrenton, USA). Differences between groups were tested with Tukey’s honest significant difference test post hoc test. Results are shown as means with standard error of the mean. Differences at *P* < 0.05 were considered significant.

## Electronic supplementary material

Supplementary materials

## Data Availability

The datasets analyzed during the current study are available from the corresponding author on reasonable request.
