# Supplementary material for: NF-κB-related decrease of glioma angiogenic potential by graphite nanoparticles and graphene oxide nanoplatelets
Source: Sci Rep. 2018 Oct 3;8:14733. doi: 10.1038/s41598-018-33179-3 (PMC6170400; doi:10.1038/s41598-018-33179-3)
Supplement: Supplementary file 1 — Supplementary materials [file 41598_2018_33179_MOESM1_ESM.pdf]

Supplementary materials

NF-κB-related decrease of glioma angiogenic potential by graphite nanoparticles and graphene oxide nanoplatelets

Mateusz Wierzbicki[1*], *Ewa Sawosz*[1], *Barbara Strojny*[1], *Sławomir Jaworski*[1], *Marta Grodzik*[1], *André Chwalibog*[2]

[1]Division of Nanobiotechnology, Warsaw University of Life Science, Ciszewskiego 8, 02-786 Warsaw, Poland

[2]Department of Veterinary and Animal Sciences, University of Copenhagen, Groennegaardsvej 3, 1870 Frederiksberg, Denmark

* Corresponding author

	A	B	C	D	E	F	G	H
1	BLANK	BLANK	PIGF	PIGF	GROα	GROα	POS	POS
2	BLANK	BLANK	RANTES	RANTES	IFN-γ	IFN-γ	POS	POS
3	BLANK	BLANK	TGF- β1	TGF- β1	IGF-1	IGF-1	NEG	NEG
4	BLANK	BLANK	TIMP-1	TIMP-1	IL-6	IL-6	NEG	NEG
5	BLANK	BLANK	TIMP-2	TIMP-2	IL-8	IL-8	Ang	Ang
6	BLANK	BLANK	THPO	THPO	Leptin	Leptin	EGF	EGF
7	NEG	NEG	VEGF	VEGF	MCP-1	MCP-1	ENA-78	ENA-78
8	POS	POS	VEGF-D	VEGF-D	PDGF-BB	PDGF-BB	bFGF	bFGF

Figure S1. Antibody array map. Ang, angiopoietin; bFGF, basic fibroblast growth factor; BLANK, blank spots without antibodies; EGF, epidermal growth factor; ENA-78, epithelial-neutrophil activating peptide; GROα, growth-regulated oncogene α (CXCL1); IFN-γ, interferon γ; IGF-1, insulin-like growth factor 1; IL-6, interleukin 6; IL-8, interleukin 8; MCP-1, monocyte chemotactic protein 1; NEG, negative controls (spots without antibodies printed with antibody dilution buffer); PDGF-BB, platelet-derived growth factor BB; PIGF, placental growth factor; POS, spot with biotin-conjugated IgG; RANTES, regulated upon activation normal T cell express sequence; TGF-β1, transforming growth factor β 1; THPO, thrombopoietin; TIMP-1, tissue inhibitor of metalloproteinases 1; TIMP-2, tissue inhibitor of metalloproteinases 2; VEGF, vascular endothelial growth factor; VEGF-D, vascular endothelial growth factor D.

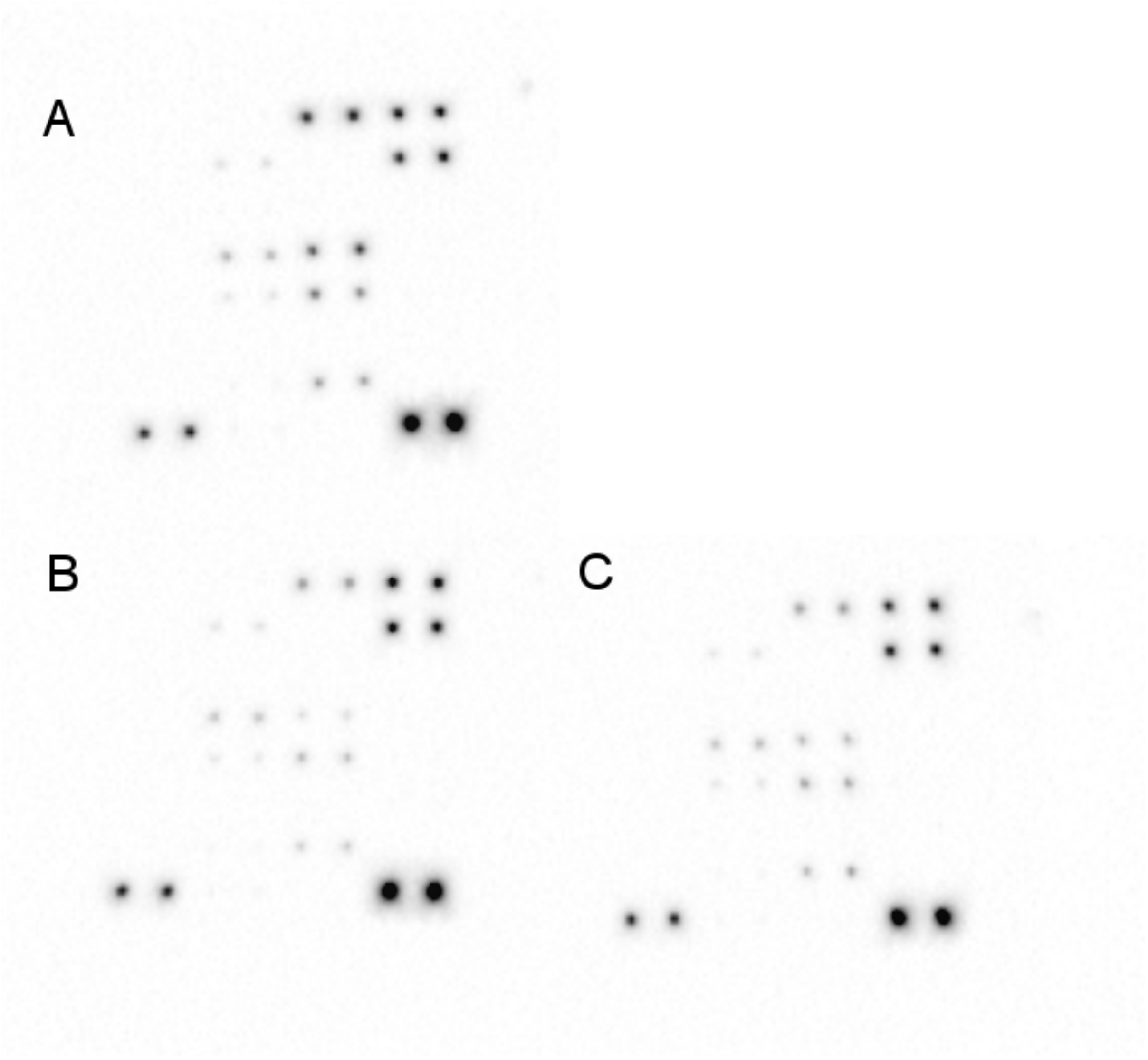

Figure S2. Uncropped antibody arrays of proangiogenic cytokine synthesis in U87 glioma cells with or without treatment (A; control) with graphite nanoparticles (B) and graphene oxide nanoplatelets (C).

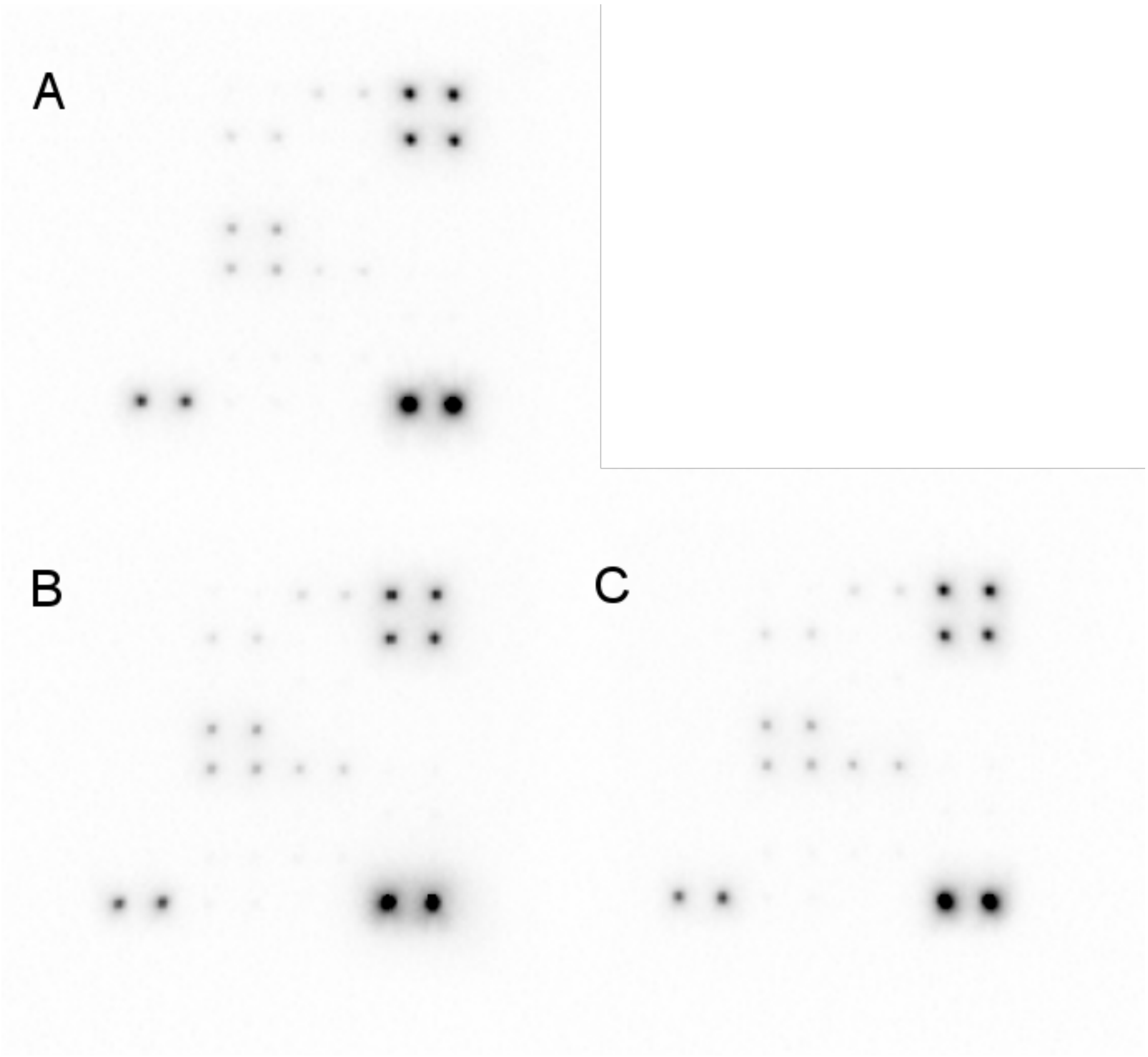

Figure S3. Uncropped antibody arrays of proangiogenic cytokine synthesis in U118 glioma cells with or without treatment (A; control) with graphite nanoparticles (B) and graphene oxide nanoplatelets (C).
